# Developmental expression patterns of neuronal growth genes across human brain stages using Bgee transcriptomic data

**DOI:** 10.1016/j.ibneur.2026.09.013

**Published:** 2026-09-19

**Authors:** Arjun Raghukumar Konijeti, Elisa Marozzi Cruz

**Affiliations:** a Cambridge Centre for International Research, Cambridge, UK; b Centre for Gene Therapy & Regenerative Medicine, King’s College London, London, UK

**Keywords:** Neuronal development, Gene expression, Brain region, GAP43, MAP1B, SEMA3B, GAL

## Abstract

Human brain maturation from prenatal life through aging is driven by tightly regulated gene expression programs supporting neuronal differentiation, axonal growth, and synaptic development. Although individual neuronal growth–related genes have been studied, limited work describes how they vary across defined human life stages and major brain regions. This study evaluated developmental expression trajectories of GAP43, MAP1B, SEMA3B, and GAL in healthy human brain tissue using central nervous system transcriptomic data from the Bgee v15 database. After excluding samples with imprecise age, unassignable whole-brain samples, and cross-accession duplicates, 433 samples were categorized into six developmental stages: late embryonic, neonate, juvenile, prime adult, late adult, and senior, and examined across forebrain and hindbrain divisions. Spearman correlation assessed relationships between expression and stage, and one-way ANOVA on log2(TPM+1) values with Tukey's post-hoc tests and Benjamini–Hochberg correction assessed differences across stages. Expression trajectories differed by gene and region. GAP43 showed the strongest age-related association, a moderate negative correlation with higher expression in earlier stages. MAP1B showed a similar but weaker downward trend. SEMA3B demonstrated a moderate positive correlation with stage, while GAL showed a weak but statistically significant decline. Forebrain regions exhibited greater developmental variability than hindbrain regions, with GAP43 and MAP1B peaking earlier in life and GAL and SEMA3B showing more modest, regionally specific variation. Neuronal growth-related genes do not follow a uniform developmental trajectory; expression patterns are gene-specific and region-dependent, highlighting temporally and anatomically distinct transcriptional programs in the healthy human brain. These findings are descriptive, and hindbrain and sex-stratified comparisons, based on small, imbalanced subgroups, are exploratory.

## 1 Introduction

From prenatal stages to late adulthood, the human brain experiences significant structural and functional development. Highly regulated, complex patterns of genomic expression guide the many changes undergone by the human brain. Processes including neuronal differentiation, axonal growth, formation of synapses, and circuit refinement are supported by these developmental gene programs, and disruptions within these programs have been found to result in neurological dysfunction and neuropsychiatric disorders (Stiles and Jernigan, 2010, Silbereis et al., 2016, Parikshak et al., 2015). Understanding the ways that neuronal growth genes are expressed across human life stages is therefore necessary for clarifying mechanisms of brain development.

There are several genes that are particularly critical in neuronal development and are clinically relevant due to their involvement in neurological disorders. Growth Associated Protein 43 (GAP43) is one of these, being associated with both synaptic plasticity and axonal growth. Altered expression of GAP43 has been associated with disorders, including Alzheimer’s disease (Benowitz and Routtenberg, 1997, Azargoonjahromi et al., 2025). Microtubule-Associated Protein 1B (MAP1B) assists in microtubule stability, axonal growth, neuronal migration, and regeneration. Neurodegenerative diseases such as epilepsy, intellectual disability, and structural brain anomalies such as cortical malformations have all been linked to pathogenic MAP1B variants (Yang et al., 2012, Archer et al., 2025). Semaphorin 3B (SEMA3B) is crucial for axon guidance and neural circuit wiring, being part of the semaphorin signaling family. Dysregulation of semaphorin pathways can imply abnormal neural connectivity and neuropsychiatric disease biology, including depression (Du et al., 2022, Liu et al., 2025). Galanin (GAL) is a neuromodulatory neuropeptide involved in neuroprotection and synaptic signaling. GAL has also been associated with stress regulation, epilepsy, and other mood-related neuropsychiatric disorders (Lang et al., 2015, Zavala-Tecuapetla et al., 2020, Barde et al., 2016). These genes are all integral components of much of the molecular machinery supporting human brain development.

GAP43, MAP1B, SEMA3B, and GAL have each been studied individually; however, a large portion of existing literature involving these genes is based on animal models, in vitro work, or analyses focused only on specific brain regions rather than the entire brain. Furthermore, there is little work done on the expression of these genes in relation to human life stages. Thus, it remains quite unclear as to how the expression of neuronal growth genes changes between embryonic, neonate, juvenile, adult, and late adult stages in the human brain.

This study examines the expression patterns of GAP43, MAP1B, SEMA3B, and GAL across defined human brain stages using data from healthy individuals from the Bgee database, a resource for cross-species genomic expression integration and developmental transcriptomics (Bastian et al., 2021). By making comparisons between various life stages, the aim of this work is to understand the importance of gene expression in neuronal growth as it relates to time of human development.

## 2 Materials and methods

### 2.1 Data source

Human brain gene expression data were obtained from Bgee v15, a curated transcriptomic database that integrates high-quality, non-redundant RNA-seq and microarray datasets across species (Bastian et al., 2021). It contains healthy wild-type expression data to provide a comparable reference of normal gene expression. Only healthy subjects without known neurological illness are included in the database. Bgee provides harmonized anatomical ontologies, standardized developmental stage annotations, and normalized expression values. Only datasets derived from Homo sapiens and annotated as central nervous system tissues were included in this analysis. Because Bgee aggregates libraries from multiple independent source studies deposited across Sequence Read Archive (SRA) and Gene Expression Omnibus (GEO) accessions, the analyzed samples originate from several sequencing platforms and experimental batches. This potential source of heterogeneity is addressed in the Limitations.

The analysis focused on four genes with established roles in neuronal development, axonal growth, and synaptic maturation: GAL (Galanin), GAP43 (Growth Associated Protein 43), SEMA3B (Semaphorin 3B), and MAP1B (Microtubule Associated Protein 1B).

### 2.2 Sample inclusion and exclusion criteria

A total of 1376 human brain samples were initially identified. Samples were then filtered in the following order: (i) samples labeled with non-specific age categories that could not be reliably mapped to precise developmental stages were excluded (n = 894; examples included “middle-aged,” “seventh decade (human),” “fifth decade stage,” and “adult stage (unspecified)”); (ii) whole-brain samples that could not be attributed to a specific brain division were excluded (n = 5); and (iii) 44 samples that had been re-deposited under a second Sequence Read Archive accession (SRP051844/SRP058181) with identical raw read counts were identified as duplicates and removed, retaining a single copy of each. The corpus callosum and striatum, which had previously been grouped with an excluded “midbrain” category, were re-classified as forebrain and retained (Supplementary Table 1). After these steps, the final analyzable dataset consisted of 433 samples.

### 2.3 Developmental stage classification

To examine developmental shifts in gene expression across major periods of human brain maturation, samples were categorized into six biologically meaningful developmental stages, selected based on human neurodevelopmental literature and Bgee metadata definitions:

1. Late Embryonic: Before birth (prenatal/late fetal period)
2. Neonate: Birth to 1 year
3. Juvenile: 1–21 years (childhood and adolescence combined)
4. Prime Adult: Ages 21–44 years (period of neural stability and peak functional integration)
5. Late Adult: Ages 45 years to 64 (period in which age-related biological changes begin to emerge)
6. Senior: Ages 65 and above

### 2.4 Brain region classification

In order to examine shifts in gene expression across various locations within the human brain, samples were categorized into two biologically important brain regions (Supplementary Table):

1. Forebrain (cortexes)
2. Hindbrain (brain stem, cerebellum, etc.)

Finer anatomical subdivision (e.g., individual cortical areas or laminae) was not performed because splitting the data into per-region, per-stage cells would yield sample sizes too small for stable estimation. Several hindbrain stages already contain only 4–7 samples. The two-division grouping was therefore chosen to preserve statistical power, and hindbrain stage-wise comparisons are treated as exploratory throughout.

### 2.5 Data extraction and preprocessing

Expression values were extracted using the Bgee data portal and programmatic interface.

Data were downloaded and subsequently processed in Microsoft Excel, including filtering, developmental category assignment, and organization by brain region. All statistical analyses were performed in Python 3.12 (SciPy and statsmodels). Because developmental stage is an ordinal variable, the relationship between transcript-per-million (TPM) expression values and developmental stage was evaluated using Spearman rank correlation. The association between expression and sex (a binary variable) was assessed using point-biserial correlation and is reported as such. Correlation analyses were conducted separately for forebrain and hindbrain samples (i.e., stratified by brain region) rather than pooled across regions.

### 2.6 Statistical analysis

Because the original dataset contained > 20 distinct brain regions, anatomically related structures were grouped to improve statistical power (Supplementary Table 1). Regions were clustered into two primary neuroanatomical divisions: Forebrain and Hindbrain. For each of the four genes (GAL, GAP43, SEMA3B, MAP1B), expression was evaluated across the six developmental stages to determine whether expression peaks occurred during embryonic, neonatal, juvenile, prime adult, late adult, or senior periods. Comparisons of expression across life stages were conducted using one-way ANOVA, performed separately for forebrain and hindbrain groupings. Prior to parametric testing, the distribution of TPM values was assessed using the Shapiro–Wilk test and examination of skewness; because RNA-seq TPM values were right-skewed and significantly non-normal, expression values were log2(TPM+1)-transformed before ANOVA. Pairwise comparisons between developmental stages followed a significant ANOVA using Tukey’s honestly significant difference (HSD) test. To account for the full set of comparisons (four genes × two regions, each with multiple pairwise contrasts), p-values were adjusted for multiple comparisons using the Benjamini–Hochberg false discovery rate (FDR) procedure. Effect sizes were reported alongside p-values (η² for omnibus ANOVA and mean differences with 95% confidence intervals for pairwise comparisons). Statistical significance was defined as p < 0.05.

Expression trajectories were visualized using trend plots across developmental stages.

Correlation plots were generated to illustrate relationships between expression and age, including associated correlation coefficients (r-values).

### 2.7 Ethical considerations

All data analyzed in this study were publicly available, fully de-identified, and obtained from Bgee in accordance with ethical standards governing secondary analysis of human biological data. No new human subjects were recruited, contacted, or involved in data generation.

## 3 Results

### 3.1 Summary of results

Across genes, correlation analyses showed differing age-related patterns, with GAP43 and MAP1B demonstrating clearer negative trends across developmental life stages, SEMA3B showing a moderate positive trend, and GAL exhibiting only modest downward change (Fig. 1). ANOVA analyses showed that forebrain expression patterns varied more prominently across stages than hindbrain. In the forebrain, GAL and SEMA3B peaked in juvenile and prime adult stages, GAP43 peaked earlier (embryonic in hindbrain and neonatal in forebrain), followed by a decline, and MAP1B showing early embryonic elevation followed by a decline across later forebrain stages. In contrast, hindbrain patterns were generally flatter, with fewer significant pairwise differences, except for consistently higher embryonic expression for GAP43 and MAP1B. SEMA3B expression appeared to increase over life stages.

**Fig. 1:**
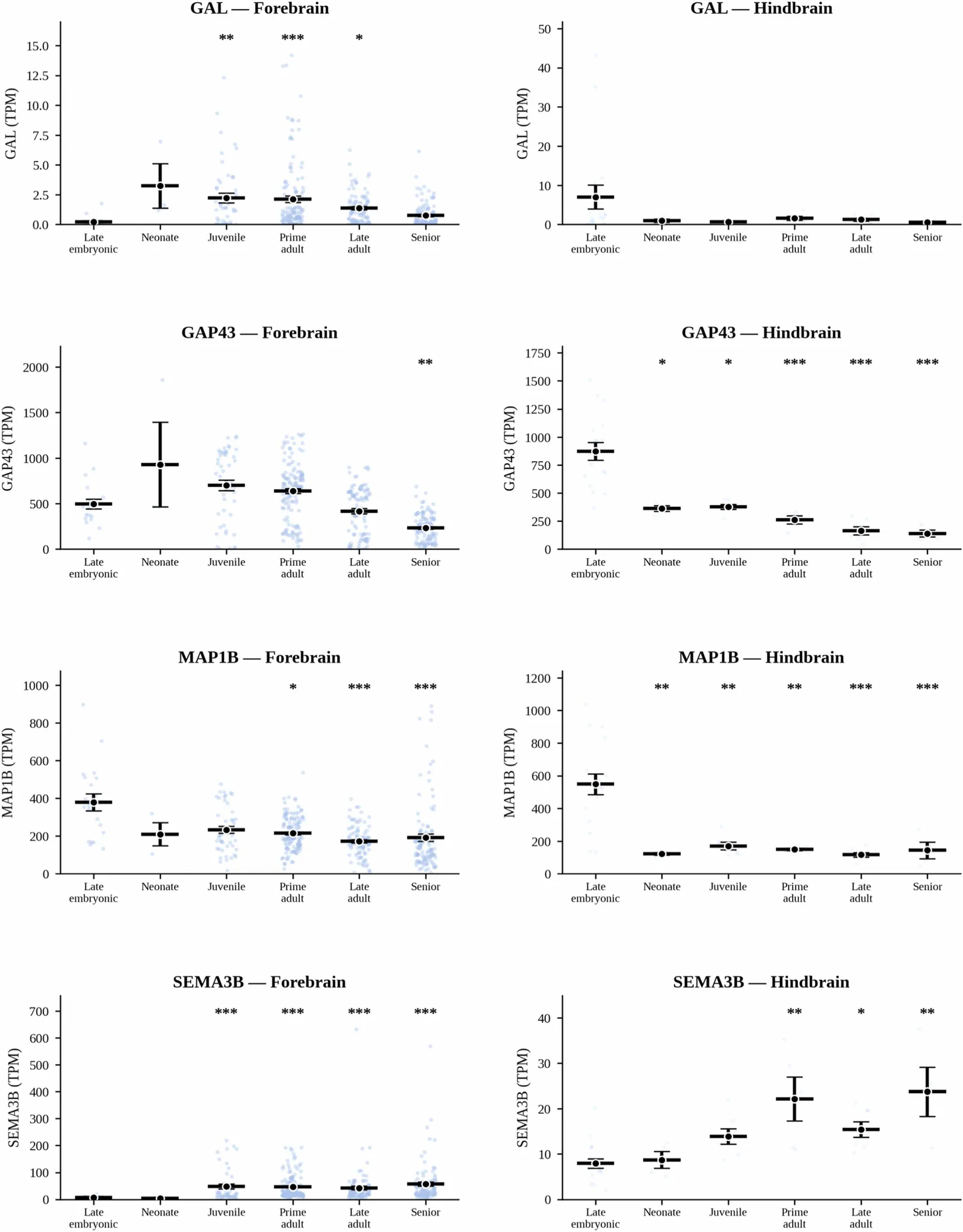
Developmental expression of GAL, GAP43, MAP1B, and SEMA3B across human brain life stages (N = 433). Expression is shown for each gene across six developmental life stages (late embryonic, neonate, juvenile, prime adult, late adult, and senior), separated by brain region (forebrain and hindbrain). Panels: (A) GAL, (B) GAP43, (C) MAP1B, and (D) SEMA3B, each shown for the forebrain (left) and hindbrain (right). Individual samples are plotted as points; the horizontal crossbar marks the group mean and the whiskers show ± the standard error of the mean (SEM), in transcript-per-million (TPM) units. The number of samples per stage and region is reported in Table 1. Asterisks denote stages that differ significantly from the late embryonic stage (one-way ANOVA on log2(TPM+1) with Tukey’s HSD post-hoc test and Benjamini–Hochberg FDR correction; *p < 0.05, **p < 0.01, ***p < 0.001). The sampling unit is an individual human brain tissue sample. All pairwise comparisons between life stages, with mean differences, 95% confidence intervals, and FDR-adjusted p-values, are reported in Supplementary Table 3. *Abbreviations: GAL (Galanin), GAP43 (Growth Associated Protein 43), MAP1B (Microtubule Associated Protein 1B), and SEMA3B (Semaphorin 3B).

**Table 1:** Subject characteristics.

**Characteristic**	**Value**
**Life stage, median age (range)**	
Late embryonic, weeks post-fertilization	–13 (–21 to –10)
Neonate, y	0 (0−0)
Juvenile, y	16 (7−21)
Prime adult, y	35 (24−44)
Late adult, y	55 (46−64)
Senior, y	78.5 (66−106)
**Sex, n (%)**	
Male	372 (85.9)
Female	61 (14.1)
**Brain region, n (%)**	
Forebrain	389 (89.8)
Hindbrain	44 (10.2)
Total analyzable samples, n	433
**Samples per developmental stage, n (forebrain / hindbrain)**	
Late embryonic	20 / 17
Neonate	3 / 4
Juvenile	47 / 7
Prime adult	127 / 5
Late adult	91 / 7
Senior	101 / 4

### 3.2 GAL expression trends

GAL expression demonstrated only a weak but statistically significant downward trend across developmental life stages (*Spearman r = −0.13, p = 0.009*). GAL expression showed essentially no relationship with sex (*point-biserial r = 0.00*) (Fig. 1A). These values indicate variable GAL expression across samples, with only small directional changes across life stages.

In the forebrain, GAL expression peaked in the juvenile and prime adult stages, with statistically significant pairwise differences observed for both with the embryonic stage. The prime adult stage also had significantly higher GAL expression compared to late adult and senior stages. In contrast, in the hindbrain, all comparisons of GAL expression were non-significant, indicating that mean expression across developmental stages appeared more similar in the hindbrain than in the forebrain.

### 3.3 GAP43 expression trends

GAP43 expression demonstrated the strongest developmental association among the genes evaluated, showing a negative monotonic trend across life stages (Spearman r = −0.49, p < 0.001; r² ≈ 0.24, indicating a modest proportion of shared variance and therefore a weak-to-moderate association) (Fig. 1B). GAP43 expression also showed a weak positive relationship with sex (point-biserial r = 0.18). These values indicate a clearer age-related pattern than seen with GAL, although variability remained present across samples.

In the forebrain, GAP43 demonstrated peak expression in the neonatal stage, followed by a progressive decline across older life stages. After FDR correction, senior expression was significantly lower than embryonic, juvenile, and prime adult expression, and late adult expression was significantly lower than prime adult expression (Fig. 1; all pairwise comparisons are reported in Supplementary Table 3). In the hindbrain, GAP43 expression peaked in the embryonic stage, where it was significantly higher than all other life stages. All other life stages had lower GAP43 expression, without significant pair-wise differences among them.

### 3.4 MAP1B expression trends

Across all samples, MAP1B showed a moderate negative monotonic association with developmental stage (Spearman r = −0.35, p < 0.001; r² ≈ 0.13; Fig. 1C). Within the forebrain, expression was highest in the embryonic stage and declined across subsequent stages (Spearman r = −0.32). Re-analysis of the complete dataset, including the embryonic samples, did not reproduce a bimodal pattern, and a quadratic term did not improve the model fit over a linear/monotonic model (p = 0.91); the monotonic coefficient therefore adequately describes the trajectory. MAP1B expression also showed weak correlations with sex (point-biserial r = −0.09), indicating that most of the patterning observed was related to age rather than these additional variables.

In the forebrain, MAP1B expression was highest in the embryonic stage and declined across subsequent developmental stages. After correction for multiple comparisons, embryonic expression was significantly higher than that of the prime adult, late adult, and senior stages. Similar to GAP43, in the hindbrain, MAP1B expression was highest in the embryonic stage, where it was also significantly higher than all other life stages. All other life stages had lower MAP1B expression without pair-wise differences among them.

### 3.5 SEMA3B expression trends

SEMA3B demonstrated a moderate positive monotonic correlation with developmental life stage (Spearman r = 0.34, p < 0.001), indicating a slight increase in expression across lifespan categories (Fig. 1D). In the hindbrain, SEMA3B increased across development, with the prime adult, late adult, and senior stages each significantly higher than the embryonic stage. Additionally, SEMA3B showed weak correlations with sex (point-biserial r = 0.12), suggesting that relationships had more variability across samples.

In the forebrain, similar to GAL expression, SEMA3B demonstrated peak expression during the juvenile and prime adult stages, with statistically significant greater expression observed for both compared with the embryonic stage. Prime adult also had significantly higher SEMA3B expression compared to late adults. In the hindbrain, SEMA3B expression gradually increased across developmental stages, reaching statistical significance for comparison between late adult and embryonic life stages.

## 4 Discussion

In this study, we used curated human brain transcriptomic data from healthy individuals in Bgee to describe developmental expression patterns of four neuronal growth-related genes (GAL, GAP43, MAP1B, and SEMA3B) across major life stages and brain regions. Overall, the results show that these genes do not follow a uniform developmental trajectory. Instead, they exhibit distinct age-related patterns that differ by gene and between forebrain and hindbrain, consistent with the idea that neuronal growth and guidance programs are both temporally and regionally specialized during human brain maturation (Stiles and Jernigan, 2010, Silbereis et al., 2016, Parikshak et al., 2015).

GAP43 and MAP1B showed the clearest negative correlations with developmental stages, with higher expression in earlier life periods and decreasing expression in later stages. This pattern aligns well with prior work demonstrating that GAP43 is highly expressed during periods of axonal growth and synaptic remodeling, and is often considered a marker of developmental plasticity and axonal sprouting (Benowitz and Routtenberg, 1997, Azargoonjahromi et al., 2025). Similarly, MAP1B has been shown to be critical for microtubule dynamics, axon extension, and neuronal migration, with prominent expression during early neurodevelopment in experimental models (Yang et al., 2012, Archer et al., 2025). The higher embryonic and early-life expression of GAP43 and MAP1B observed in our analyses, particularly in the hindbrain where embryonic levels were consistently greater than those in later stages, is therefore broadly consistent with their established roles in early structural wiring of the nervous system. Our findings extend this prior work by providing a descriptive, stage-based view of these patterns in human brain transcriptomic data across multiple developmental periods.

By contrast, GAL and SEMA3B demonstrated more modest and regionally restricted developmental variation. GAL showed only a weak negative correlation with development, with relatively small directional changes across life stages. This is consistent with its known role as a neuromodulatory peptide that is expressed in multiple ascending pathways and implicated in synaptic signaling, stress responses, and neuroprotection rather than being restricted to early developmental windows (Lang et al., 2015). In our data, forebrain GAL expression peaked in juvenile and prime adult stages, whereas hindbrain GAL expression showed no significant pairwise differences between stages, suggesting that GAL may be more involved in regionally specific modulation rather than early-developmental growth or hormonal patterns.

SEMA3B, a member of the semaphorin family involved in axon guidance and neuronal connectivity (Du et al., 2022, Liu et al., 2025), showed a weak positive correlation with developmental stage and demonstrated forebrain peaks in juvenile and prime adult periods. Semaphorin signaling has been implicated in both early patterning and later circuit refinement, and dysregulation of axon guidance pathways has been linked to neuropsychiatric and neurodegenerative disorders (Du et al., 2022, Liu et al., 2025). The gradual increase in hindbrain SEMA3B expression across development, with higher expression in late adulthood relative to embryonic stages, is compatible with the idea that semaphorin-related signaling can persist beyond early embryogenesis and may contribute to ongoing structural or synaptic adjustments in later life. Our observations do not establish causality but suggest that semaphorin-related developmental changes are detectable at the transcript level in the human brain across broad life stages.

Several of the genes examined here have been implicated in neurological and neurodevelopmental disorders, which provides additional context for the observed patterns. GAP43 alterations have been reported in neurodegenerative conditions such as Alzheimer’s disease mild cognitive impairment (Benowitz and Routtenberg, 1997, Azargoonjahromi et al., 2025). A recent cross-sectional study by Azargoonjahromi et al. found that cerebrospinal fluid (CSF) GAP43 levels were highest in the Alzheimer’s dementia (AD) group, followed by the mild cognitive impairment (MCI) group, and lowest in the cognitively normal (CN) group (Azargoonjahromi et al., 2025). Elevated CSF GAP43 levels also positively correlated with Aβ accumulation in individuals with MCI. In contrast, elevated CSF GAP43 levels were associated with reduced Aβ accumulation in individuals with Alzheimer’s dementia, potentially indicating a dynamic and dual role of GAP43 in neurodegeneration. In our study, we found a weak-to-moderate negative association (Spearman r = −0.49) with peak expression in the embryonic and neonate stages. The low GAP43 expression in adult stages in our cohort would correspond to that seen in the CN group in Azargoonjahromi’s study.

MAP1B is a critical regulator of cytoskeletal dynamics, axonal growth, and neuronal migration during central nervous system development, and experimental studies have highlighted its essential role in shaping early neural architecture (Yang et al., 2012). More recent genetic evidence from a case series found that individuals with pathogenic loss-of-function MAP1B variants exhibited global developmental delay, intellectual disability, behavioral dysregulation, and focal epilepsy, reinforcing the importance of appropriate MAP1B expression during critical developmental windows (Archer et al., 2025). Within this context, our findings of a moderate negative correlation between MAP1B expression and developmental life stage suggest biologically coherent developmental downregulation, consistent with MAP1B’s primary role early in brain formation. Regionally, the high embryonic expression in both forebrain and hindbrain aligns with its function in early neuronal growth and migration, while the largely lower and more stable expression across later stages suggests diminished reliance on MAP1B as structural maturation stabilizes. Notably, re-analysis of the complete dataset, including the embryonic samples, showed that forebrain MAP1B expression was highest in the embryonic stage and decreased across later stages, reaching its lowest levels in the late adult and senior stages. An earlier impression of a bimodal trajectory with secondary re-elevation in seniors was not reproduced once the full dataset was analysed, and a quadratic model offered no improvement over a linear (monotonic) fit. This monotonic developmental decline is consistent with MAP1B’s established role in axon extension and neuronal migration during early brain formation and with a diminished structural requirement for MAP1B once neural architecture stabilizes.

Semaphorin pathway genes, including class 3 semaphorins, have been linked to changes in synaptic plasticity that can be associated with depression, hyperalgesia, and allodynia (Du et al., 2022, Liu et al., 2025). Liu et al. recently demonstrated that inactivating SEMA3B or its axonal receptor components in mice led to incomplete unmyelinated axon separation and envelopment by Schwann cells (SC), eliciting hyperalgesia and allodynia (Liu et al., 2025). Conversely, increasing SEMA3B levels in SCs accelerated axon separation and envelopment, alleviating these neuropathic conditions. Although our analyses do not evaluate disease cohorts or outcomes, the developmental expression trajectories described here may help frame hypotheses about when disruptions in these genes could be most impactful during human brain maturation.

Prior work has linked alterations in GAL pathways to neurological and psychiatric disease states, including epilepsy and major depressive disorder (Zavala-Tecuapetla et al., 2020, Barde et al., 2016). In epilepsy models, GAL receptor agonists demonstrate anticonvulsant efficacy and neuroprotective activity, due to its ability to regulate neuronal hyperexcitability, although effects may vary by brain region and developmental context (Zavala-Tecuapetla et al., 2020). These observations provide context for our descriptive finding that forebrain GAL expression peaked during the juvenile and prime adult stages; we note only that this temporal pattern is compatible with a role for GAL in excitability modulation, without implying that the present data establish any functional or therapeutic relationship. Similarly, altered GAL transcript levels and epigenetic regulation have been reported in limbic regions in major depressive disorder, underscoring the relevance of region-specific GAL expression to brain function and disease vulnerability (Barde et al., 2016). Within this framework, our data showed a more pronounced and stage-specific elevation in forebrain GAL expression during juvenile and prime adult periods, which may reflect temporally bounded functional demands in cortical and limbic networks, consistent with GAL’s proposed roles in stress adaptation and excitability modulation. Collectively, these findings emphasize that GAL biology is shaped by both anatomical and developmental context, reinforcing the importance of region-specific analyses when interpreting GAL function and its potential relevance to neurological disorders.

This work has several limitations. First, the analyses rely on existing transcriptomic datasets compiled in Bgee, which may have uneven sampling across brain regions and life stages and do not resolve cell-type–specific patterns (Bastian et al., 2021). Second, because Bgee aggregates libraries from multiple independent source studies, sequencing platforms, and SRA/GEO accessions, residual batch or dataset-of-origin effects cannot be excluded despite Bgee’s harmonized curation and normalization. We did not perform explicit batch-effect modeling. Third, expression was summarized at the regional level and grouped into broad forebrain and hindbrain categories to improve statistical efficiency. Finer-grained anatomical or laminar analyses were beyond the scope of the current dataset. Fourth, potential influencing factors other than developmental stage were considered but not exhaustively modeled. Brain region was handled by conducting all analyses stratified by forebrain versus hindbrain, and sex was assessed with point-biserial correlations, but the male-predominant composition (85.9%) meant the dataset was not adequately powered for sex-stratified analysis. Fifth, the dataset was substantially imbalanced: forebrain samples greatly outnumbered hindbrain samples (389 vs. 44), so hindbrain stage-wise comparisons rest on small per-cell sample sizes (n = 4–7 at several stages) and are framed as exploratory. Sixth, 894 of 1376 samples (65%) were excluded because of imprecise age annotation, which may introduce selection bias; a formal comparison of excluded versus retained samples was not performed. Collectively, these limitations reinforce that the present results represent descriptive associations and do not support causal or disease-mechanistic conclusions.

Despite these limitations, this study provides a structured overview of how four neuronal growth–related genes are expressed across human brain developmental stages, highlighting both shared and distinct features across genes and regions. The observation that GAP43 and MAP1B show stronger early-life expression, while GAL and SEMA3B exhibit more modest and region-specific developmental changes, suggests that different components of the neuronal growth and guidance machinery may operate on partially overlapping but distinct temporal schedules. Future work integrating cell-type–specific transcriptomics, longitudinal developmental datasets, and disease cohorts may help clarify how these developmental trajectories relate to neural circuit formation and to the timing and vulnerability of neurodevelopmental and neurodegenerative disorders.

## 5 Conclusions

In summary, our study provides a descriptive, stage-oriented view of neuronal growth gene expression in the healthy human brain, a perspective that has been relatively underrepresented in literature, primarily concerning animal models and region-specific studies. Future work that integrates single-cell transcriptomics, longitudinal studies, and disease cohorts will be essential to determine how deviations from normal developmental trajectories contribute to neurodevelopmental and neurodegenerative conditions across the lifespan.

## CRediT authorship contribution statement

**Arjun Raghukumar Konijeti:** Writing – review & editing, Writing – original draft, Formal analysis, Data curation, Conceptualization. **Elisa Marozzi Cruz:** Writing – review & editing, Supervision, Software, Project administration, Methodology, Investigation, Formal analysis, Data curation, Conceptualization.

## Declaration of Generative AI and AI-assisted technologies in the writing process

Statement: During the preparation of this work the author(s) used OpenEvidence to improve the readability and organization of the manuscript text, and assist in developing and formatting the figure and tables. After using this tool/service, the author(s) reviewed and edited the content as needed and take(s) full responsibility for the content of the published article.

## Conflicts of Interest

The authors declare that they have no known competing financial interests or personal relationships that could have appeared to influence the work reported in this paper.
