# Supplementary material for: Developmental expression patterns of neuronal growth genes across human brain stages using Bgee transcriptomic data
Source: IBRO Neurosci Rep. 2026 Sep 19;21:841–7. doi: 10.1016/j.ibneur.2026.09.013 (PMC13632171; doi:10.1016/j.ibneur.2026.09.013)
Supplement: Supplementary file 1 [file mmc1.docx]

# Supplementary Material

Re: Manuscript IBNEUR-D-26-00285 (IBRO Neuroscience Reports)

## Supplementary Table 1. Classification of Brain Regions (corrected)

| **Brain region (Bgee label)** | **Classification for analyses** | **Status** |
| --- | --- | --- |
| Brain (whole brain) | Excluded ‡ | Excluded ‡ |
| Frontal Cortex | Forebrain | — |
| Prefrontal Cortex | Forebrain | — |
| Temporal Lobe | Forebrain | — |
| Cerebellum | Hindbrain | — |
| Corpus Callosum | Forebrain § | Corrected |
| Precuneus Cortex | Forebrain | — |
| Dorsolateral Prefrontal Cortex | Forebrain | — |
| Forebrain | Forebrain | — |
| Hindbrain | Hindbrain | — |
| Telencephalon | Forebrain | — |
| Fusiform Gyrus | Forebrain | — |
| Premotor Cortex | Forebrain | — |
| Primary Visual Cortex | Forebrain | — |
| Anterior Cingulate Cortex | Forebrain | — |
| Striatum | Forebrain § | Corrected |
| Ammon's Horn | Forebrain | — |
| Brodmann (1909) areas 6, 9, 21, 22, 39, 40, 44–47 | Forebrain (neocortex) | — |

*§ Corrected: corpus callosum is telencephalic commissural white matter (forebrain); the striatum is basal ganglia (forebrain). These 10 samples were previously grouped as the excluded “midbrain” and are now correctly classified as forebrain and retained. The analyzable N is 433, reached from 1,376 initial samples after excluding 894 with imprecise age annotation, excluding 5 whole-brain samples, and removing 44 duplicate re-deposited samples (the previously reported figure was 471).*

*‡ “Brain” denotes whole-brain samples (n=5), which cannot be assigned to a single division and were excluded.*

## Supplementary Table 2. Group Summary Statistics (TPM)

| **Gene** | **Region** | **Stage** | **n** | **Mean ± SD** | **Median (IQR)** |
| --- | --- | --- | --- | --- | --- |
| GAL | Forebrain | Late embryonic | 20 | 0.2 ± 0.4 | 0.1 (0.0–0.2) |
| GAL | Forebrain | Neonate | 3 | 3.2 ± 3.2 | 1.6 (1.4–4.3) |
| GAL | Forebrain | Juvenile | 47 | 2.2 ± 2.8 | 1.3 (0.0–3.3) |
| GAL | Forebrain | Prime adult | 127 | 2.1 ± 2.9 | 1.0 (0.3–2.7) |
| GAL | Forebrain | Late adult | 91 | 1.4 ± 1.3 | 1.1 (0.3–1.9) |
| GAL | Forebrain | Senior | 101 | 0.8 ± 1.0 | 0.3 (0.1–1.1) |
| GAL | Hindbrain | Late embryonic | 17 | 7.0 ± 12.6 | 1.9 (0.6–2.9) |
| GAL | Hindbrain | Neonate | 4 | 0.9 ± 0.9 | 0.7 (0.3–1.3) |
| GAL | Hindbrain | Juvenile | 7 | 0.6 ± 0.4 | 0.6 (0.5–0.7) |
| GAL | Hindbrain | Prime adult | 5 | 1.6 ± 1.2 | 1.2 (1.0–1.4) |
| GAL | Hindbrain | Late adult | 7 | 1.2 ± 1.0 | 0.8 (0.6–2.0) |
| GAL | Hindbrain | Senior | 4 | 0.6 ± 0.7 | 0.4 (0.1–0.8) |
| GAP43 | Forebrain | Late embryonic | 20 | 495.8 ± 239.5 | 471.5 (353.4–542.5) |
| GAP43 | Forebrain | Neonate | 3 | 928.5 ± 803.8 | 464.7 (464.4–1160.7) |
| GAP43 | Forebrain | Juvenile | 47 | 701.1 ± 396.2 | 797.0 (382.8–1054.0) |
| GAP43 | Forebrain | Prime adult | 127 | 638.7 ± 318.6 | 684.5 (436.9–813.4) |
| GAP43 | Forebrain | Late adult | 91 | 416.8 ± 264.9 | 452.2 (159.2–626.7) |
| GAP43 | Forebrain | Senior | 101 | 233.5 ± 151.3 | 224.9 (106.5–331.6) |
| GAP43 | Hindbrain | Late embryonic | 17 | 872.3 ± 330.0 | 850.4 (650.9–1057.4) |
| GAP43 | Hindbrain | Neonate | 4 | 363.0 ± 49.2 | 374.5 (351.2–386.3) |
| GAP43 | Hindbrain | Juvenile | 7 | 375.9 ± 58.2 | 370.2 (358.9–415.1) |
| GAP43 | Hindbrain | Prime adult | 5 | 261.6 ± 76.9 | 293.4 (227.8–310.0) |
| GAP43 | Hindbrain | Late adult | 7 | 165.6 ± 95.7 | 126.2 (104.6–244.1) |
| GAP43 | Hindbrain | Senior | 4 | 139.6 ± 62.8 | 124.4 (89.9–174.1) |
| MAP1B | Forebrain | Late embryonic | 20 | 378.6 ± 199.7 | 356.8 (206.7–508.1) |
| MAP1B | Forebrain | Neonate | 3 | 210.5 ± 107.5 | 207.7 (156.1–263.5) |
| MAP1B | Forebrain | Juvenile | 47 | 233.9 ± 123.1 | 204.9 (139.9–318.4) |
| MAP1B | Forebrain | Prime adult | 127 | 215.6 ± 88.1 | 206.6 (154.7–278.5) |
| MAP1B | Forebrain | Late adult | 91 | 173.7 ± 81.4 | 174.0 (129.5–216.6) |
| MAP1B | Forebrain | Senior | 101 | 191.8 ± 190.7 | 128.5 (79.0–221.0) |
| MAP1B | Hindbrain | Late embryonic | 17 | 548.8 ± 261.6 | 545.0 (399.6–631.2) |
| MAP1B | Hindbrain | Neonate | 4 | 122.6 ± 15.5 | 125.8 (117.4–131.0) |
| MAP1B | Hindbrain | Juvenile | 7 | 170.9 ± 61.0 | 157.0 (125.6–192.6) |
| MAP1B | Hindbrain | Prime adult | 5 | 149.4 ± 17.3 | 144.7 (141.6–163.2) |
| MAP1B | Hindbrain | Late adult | 7 | 116.7 ± 36.7 | 109.1 (99.8–134.7) |
| MAP1B | Hindbrain | Senior | 4 | 144.5 ± 101.0 | 130.9 (71.3–204.1) |
| SEMA3B | Forebrain | Late embryonic | 20 | 7.2 ± 3.6 | 6.5 (5.0–8.7) |
| SEMA3B | Forebrain | Neonate | 3 | 4.4 ± 1.3 | 3.8 (3.7–4.9) |
| SEMA3B | Forebrain | Juvenile | 47 | 48.1 ± 60.6 | 19.3 (10.3–51.6) |
| SEMA3B | Forebrain | Prime adult | 127 | 46.8 ± 45.6 | 25.2 (15.8–63.3) |
| SEMA3B | Forebrain | Late adult | 91 | 42.5 ± 71.2 | 26.6 (15.1–44.5) |
| SEMA3B | Forebrain | Senior | 101 | 56.6 ± 74.0 | 35.2 (18.6–64.4) |
| SEMA3B | Hindbrain | Late embryonic | 17 | 7.9 ± 4.4 | 7.2 (6.0–8.2) |
| SEMA3B | Hindbrain | Neonate | 4 | 8.7 ± 3.7 | 8.7 (5.7–11.6) |
| SEMA3B | Hindbrain | Juvenile | 7 | 13.9 ± 4.6 | 12.4 (11.0–16.1) |
| SEMA3B | Hindbrain | Prime adult | 5 | 22.1 ± 10.8 | 23.6 (11.4–29.4) |
| SEMA3B | Hindbrain | Late adult | 7 | 15.4 ± 4.6 | 13.5 (11.9–19.6) |
| SEMA3B | Hindbrain | Senior | 4 | 23.7 ± 10.9 | 23.1 (18.2–28.6) |

*Values in TPM. Cells with very small n (e.g., several hindbrain stages) should be interpreted with caution.*

## Supplementary Table 3. All Pairwise Comparisons Between Life Stages

Every pairwise contrast between developmental stages, for each gene and brain region (120 comparisons in total). Comparisons were made by one-way ANOVA on log₂(TPM+1) with Tukey’s HSD post-hoc test; p-values were then adjusted across the full family of 120 contrasts using the Benjamini–Hochberg false discovery rate (FDR) procedure. Mean differences and 95% confidence intervals are on the log₂ scale (Stage B minus Stage A). Significance is based on the FDR-adjusted p-value: *p < 0.05, **p < 0.01, ***p < 0.001; ns = not significant. Of the 120 comparisons, 36 remained significant after correction.

| **Gene** | **Region** | **Stage A** | **Stage B** | **Mean diff. (log₂)** | **95% CI** | **p (raw)** | **p (FDR)** | **Sig.** |
| --- | --- | --- | --- | --- | --- | --- | --- | --- |
| GAL | Forebrain | Late embryonic | Neonate | +1.58 | (+0.03, +3.13) | 0.043 | 0.119 | ns |
| GAL | Forebrain | Late embryonic | Juvenile | +1.01 | (+0.34, +1.68) | 3.0e-04 | 0.002 | ****** |
| GAL | Forebrain | Late embryonic | Prime adult | +0.96 | (+0.36, +1.57) | 1.0e-04 | 7.5e-04 | ******* |
| GAL | Forebrain | Late embryonic | Late adult | +0.80 | (+0.18, +1.42) | 0.003 | 0.013 | ***** |
| GAL | Forebrain | Late embryonic | Senior | +0.40 | (-0.21, +1.01) | 0.423 | 0.847 | ns |
| GAL | Forebrain | Neonate | Juvenile | -0.57 | (-2.06, +0.92) | 0.882 | 1.000 | ns |
| GAL | Forebrain | Neonate | Prime adult | -0.62 | (-2.08, +0.85) | 0.833 | 1.000 | ns |
| GAL | Forebrain | Neonate | Late adult | -0.78 | (-2.25, +0.69) | 0.651 | 1.000 | ns |
| GAL | Forebrain | Neonate | Senior | -1.18 | (-2.65, +0.29) | 0.194 | 0.431 | ns |
| GAL | Forebrain | Juvenile | Prime adult | -0.05 | (-0.47, +0.38) | 1.000 | 1.000 | ns |
| GAL | Forebrain | Juvenile | Late adult | -0.21 | (-0.66, +0.24) | 0.768 | 1.000 | ns |
| GAL | Forebrain | Juvenile | Senior | -0.61 | (-1.05, -0.17) | 0.001 | 0.006 | ****** |
| GAL | Forebrain | Prime adult | Late adult | -0.16 | (-0.51, +0.18) | 0.753 | 1.000 | ns |
| GAL | Forebrain | Prime adult | Senior | -0.56 | (-0.90, -0.23) | 0.0e+00 | 0.0e+00 | ******* |
| GAL | Forebrain | Late adult | Senior | -0.40 | (-0.76, -0.04) | 0.020 | 0.061 | ns |
| GAL | Hindbrain | Late embryonic | Neonate | -1.04 | (-2.95, +0.87) | 0.581 | 1.000 | ns |
| GAL | Hindbrain | Late embryonic | Juvenile | -1.24 | (-2.78, +0.31) | 0.181 | 0.409 | ns |
| GAL | Hindbrain | Late embryonic | Prime adult | -0.60 | (-2.35, +1.15) | 0.903 | 1.000 | ns |
| GAL | Hindbrain | Late embryonic | Late adult | -0.86 | (-2.40, +0.69) | 0.560 | 1.000 | ns |
| GAL | Hindbrain | Late embryonic | Senior | -1.35 | (-3.26, +0.56) | 0.300 | 0.629 | ns |
| GAL | Hindbrain | Neonate | Juvenile | -0.20 | (-2.35, +1.96) | 1.000 | 1.000 | ns |
| GAL | Hindbrain | Neonate | Prime adult | +0.44 | (-1.87, +2.75) | 0.992 | 1.000 | ns |
| GAL | Hindbrain | Neonate | Late adult | +0.18 | (-1.97, +2.34) | 1.000 | 1.000 | ns |
| GAL | Hindbrain | Neonate | Senior | -0.31 | (-2.74, +2.12) | 0.999 | 1.000 | ns |
| GAL | Hindbrain | Juvenile | Prime adult | +0.63 | (-1.38, +2.65) | 0.932 | 1.000 | ns |
| GAL | Hindbrain | Juvenile | Late adult | +0.38 | (-1.46, +2.22) | 0.989 | 1.000 | ns |
| GAL | Hindbrain | Juvenile | Senior | -0.11 | (-2.27, +2.04) | 1.000 | 1.000 | ns |
| GAL | Hindbrain | Prime adult | Late adult | -0.26 | (-2.27, +1.76) | 0.999 | 1.000 | ns |
| GAL | Hindbrain | Prime adult | Senior | -0.75 | (-3.05, +1.56) | 0.925 | 1.000 | ns |
| GAL | Hindbrain | Late adult | Senior | -0.49 | (-2.65, +1.67) | 0.983 | 1.000 | ns |
| GAP43 | Forebrain | Late embryonic | Neonate | +0.73 | (-1.82, +3.28) | 0.964 | 1.000 | ns |
| GAP43 | Forebrain | Late embryonic | Juvenile | +0.01 | (-1.09, +1.10) | 1.000 | 1.000 | ns |
| GAP43 | Forebrain | Late embryonic | Prime adult | +0.24 | (-0.75, +1.23) | 0.982 | 1.000 | ns |
| GAP43 | Forebrain | Late embryonic | Late adult | -0.73 | (-1.75, +0.28) | 0.304 | 0.629 | ns |
| GAP43 | Forebrain | Late embryonic | Senior | -1.34 | (-2.35, -0.34) | 0.002 | 0.010 | ****** |
| GAP43 | Forebrain | Neonate | Juvenile | -0.72 | (-3.17, +1.73) | 0.959 | 1.000 | ns |
| GAP43 | Forebrain | Neonate | Prime adult | -0.49 | (-2.89, +1.92) | 0.992 | 1.000 | ns |
| GAP43 | Forebrain | Neonate | Late adult | -1.46 | (-3.88, +0.95) | 0.508 | 0.987 | ns |
| GAP43 | Forebrain | Neonate | Senior | -2.07 | (-4.48, +0.34) | 0.138 | 0.337 | ns |
| GAP43 | Forebrain | Juvenile | Prime adult | +0.24 | (-0.47, +0.94) | 0.929 | 1.000 | ns |
| GAP43 | Forebrain | Juvenile | Late adult | -0.74 | (-1.48, -0.00) | 0.049 | 0.124 | ns |
| GAP43 | Forebrain | Juvenile | Senior | -1.35 | (-2.08, -0.62) | 0.0e+00 | 0.0e+00 | ******* |
| GAP43 | Forebrain | Prime adult | Late adult | -0.98 | (-1.54, -0.41) | 0.0e+00 | 0.0e+00 | ******* |
| GAP43 | Forebrain | Prime adult | Senior | -1.59 | (-2.13, -1.04) | 0.0e+00 | 0.0e+00 | ******* |
| GAP43 | Forebrain | Late adult | Senior | -0.61 | (-1.20, -0.01) | 0.042 | 0.119 | ns |
| GAP43 | Hindbrain | Late embryonic | Neonate | -1.17 | (-2.18, -0.17) | 0.015 | 0.050 | ***** |
| GAP43 | Hindbrain | Late embryonic | Juvenile | -1.13 | (-1.94, -0.31) | 0.002 | 0.010 | ***** |
| GAP43 | Hindbrain | Late embryonic | Prime adult | -1.69 | (-2.61, -0.77) | 0.0e+00 | 0.0e+00 | ******* |
| GAP43 | Hindbrain | Late embryonic | Late adult | -2.54 | (-3.36, -1.73) | 0.0e+00 | 0.0e+00 | ******* |
| GAP43 | Hindbrain | Late embryonic | Senior | -2.64 | (-3.64, -1.63) | 0.0e+00 | 0.0e+00 | ******* |
| GAP43 | Hindbrain | Neonate | Juvenile | +0.05 | (-1.09, +1.18) | 1.000 | 1.000 | ns |
| GAP43 | Hindbrain | Neonate | Prime adult | -0.52 | (-1.74, +0.69) | 0.790 | 1.000 | ns |
| GAP43 | Hindbrain | Neonate | Late adult | -1.37 | (-2.51, -0.24) | 0.010 | 0.037 | ***** |
| GAP43 | Hindbrain | Neonate | Senior | -1.47 | (-2.75, -0.19) | 0.017 | 0.054 | ns |
| GAP43 | Hindbrain | Juvenile | Prime adult | -0.57 | (-1.63, +0.49) | 0.602 | 1.000 | ns |
| GAP43 | Hindbrain | Juvenile | Late adult | -1.42 | (-2.38, -0.45) | 0.001 | 0.005 | ****** |
| GAP43 | Hindbrain | Juvenile | Senior | -1.51 | (-2.65, -0.38) | 0.004 | 0.014 | ***** |
| GAP43 | Hindbrain | Prime adult | Late adult | -0.85 | (-1.91, +0.21) | 0.179 | 0.409 | ns |
| GAP43 | Hindbrain | Prime adult | Senior | -0.95 | (-2.16, +0.27) | 0.204 | 0.445 | ns |
| GAP43 | Hindbrain | Late adult | Senior | -0.10 | (-1.23, +1.04) | 1.000 | 1.000 | ns |
| MAP1B | Forebrain | Late embryonic | Neonate | -0.79 | (-2.50, +0.91) | 0.768 | 1.000 | ns |
| MAP1B | Forebrain | Late embryonic | Juvenile | -0.74 | (-1.48, -0.01) | 0.046 | 0.121 | ns |
| MAP1B | Forebrain | Late embryonic | Prime adult | -0.76 | (-1.42, -0.09) | 0.015 | 0.050 | ***** |
| MAP1B | Forebrain | Late embryonic | Late adult | -1.15 | (-1.83, -0.47) | 0.0e+00 | 0.0e+00 | ******* |
| MAP1B | Forebrain | Late embryonic | Senior | -1.32 | (-2.00, -0.65) | 0.0e+00 | 0.0e+00 | ******* |
| MAP1B | Forebrain | Neonate | Juvenile | +0.05 | (-1.59, +1.69) | 1.000 | 1.000 | ns |
| MAP1B | Forebrain | Neonate | Prime adult | +0.04 | (-1.57, +1.64) | 1.000 | 1.000 | ns |
| MAP1B | Forebrain | Neonate | Late adult | -0.36 | (-1.98, +1.25) | 0.988 | 1.000 | ns |
| MAP1B | Forebrain | Neonate | Senior | -0.53 | (-2.14, +1.08) | 0.934 | 1.000 | ns |
| MAP1B | Forebrain | Juvenile | Prime adult | -0.01 | (-0.48, +0.46) | 1.000 | 1.000 | ns |
| MAP1B | Forebrain | Juvenile | Late adult | -0.41 | (-0.91, +0.08) | 0.163 | 0.390 | ns |
| MAP1B | Forebrain | Juvenile | Senior | -0.58 | (-1.07, -0.10) | 0.009 | 0.033 | ***** |
| MAP1B | Forebrain | Prime adult | Late adult | -0.40 | (-0.78, -0.02) | 0.032 | 0.094 | ns |
| MAP1B | Forebrain | Prime adult | Senior | -0.57 | (-0.94, -0.20) | 2.0e-04 | 0.001 | ****** |
| MAP1B | Forebrain | Late adult | Senior | -0.17 | (-0.57, +0.23) | 0.825 | 1.000 | ns |
| MAP1B | Hindbrain | Late embryonic | Neonate | -1.96 | (-3.13, -0.79) | 2.0e-04 | 0.001 | ****** |
| MAP1B | Hindbrain | Late embryonic | Juvenile | -1.54 | (-2.49, -0.60) | 2.0e-04 | 0.001 | ****** |
| MAP1B | Hindbrain | Late embryonic | Prime adult | -1.68 | (-2.74, -0.61) | 4.0e-04 | 0.002 | ****** |
| MAP1B | Hindbrain | Late embryonic | Late adult | -2.09 | (-3.03, -1.14) | 0.0e+00 | 0.0e+00 | ******* |
| MAP1B | Hindbrain | Late embryonic | Senior | -2.02 | (-3.19, -0.85) | 1.0e-04 | 7.5e-04 | ******* |
| MAP1B | Hindbrain | Neonate | Juvenile | +0.42 | (-0.90, +1.73) | 0.932 | 1.000 | ns |
| MAP1B | Hindbrain | Neonate | Prime adult | +0.28 | (-1.12, +1.69) | 0.990 | 1.000 | ns |
| MAP1B | Hindbrain | Neonate | Late adult | -0.13 | (-1.44, +1.19) | 1.000 | 1.000 | ns |
| MAP1B | Hindbrain | Neonate | Senior | -0.06 | (-1.55, +1.42) | 1.000 | 1.000 | ns |
| MAP1B | Hindbrain | Juvenile | Prime adult | -0.13 | (-1.36, +1.10) | 1.000 | 1.000 | ns |
| MAP1B | Hindbrain | Juvenile | Late adult | -0.54 | (-1.66, +0.58) | 0.700 | 1.000 | ns |
| MAP1B | Hindbrain | Juvenile | Senior | -0.48 | (-1.79, +0.84) | 0.884 | 1.000 | ns |
| MAP1B | Hindbrain | Prime adult | Late adult | -0.41 | (-1.64, +0.82) | 0.915 | 1.000 | ns |
| MAP1B | Hindbrain | Prime adult | Senior | -0.34 | (-1.75, +1.06) | 0.976 | 1.000 | ns |
| MAP1B | Hindbrain | Late adult | Senior | +0.07 | (-1.25, +1.38) | 1.000 | 1.000 | ns |
| SEMA3B | Forebrain | Late embryonic | Neonate | -0.52 | (-2.77, +1.72) | 0.985 | 1.000 | ns |
| SEMA3B | Forebrain | Late embryonic | Juvenile | +1.75 | (+0.78, +2.72) | 0.0e+00 | 0.0e+00 | ******* |
| SEMA3B | Forebrain | Late embryonic | Prime adult | +2.10 | (+1.22, +2.97) | 0.0e+00 | 0.0e+00 | ******* |
| SEMA3B | Forebrain | Late embryonic | Late adult | +1.87 | (+0.98, +2.77) | 0.0e+00 | 0.0e+00 | ******* |
| SEMA3B | Forebrain | Late embryonic | Senior | +2.28 | (+1.39, +3.17) | 0.0e+00 | 0.0e+00 | ******* |
| SEMA3B | Forebrain | Neonate | Juvenile | +2.28 | (+0.12, +4.44) | 0.032 | 0.094 | ns |
| SEMA3B | Forebrain | Neonate | Prime adult | +2.62 | (+0.50, +4.74) | 0.006 | 0.023 | ***** |
| SEMA3B | Forebrain | Neonate | Late adult | +2.40 | (+0.27, +4.53) | 0.017 | 0.054 | ns |
| SEMA3B | Forebrain | Neonate | Senior | +2.80 | (+0.68, +4.93) | 0.003 | 0.011 | ***** |
| SEMA3B | Forebrain | Juvenile | Prime adult | +0.34 | (-0.28, +0.96) | 0.607 | 1.000 | ns |
| SEMA3B | Forebrain | Juvenile | Late adult | +0.12 | (-0.53, +0.77) | 0.995 | 1.000 | ns |
| SEMA3B | Forebrain | Juvenile | Senior | +0.53 | (-0.11, +1.17) | 0.175 | 0.409 | ns |
| SEMA3B | Forebrain | Prime adult | Late adult | -0.22 | (-0.72, +0.28) | 0.795 | 1.000 | ns |
| SEMA3B | Forebrain | Prime adult | Senior | +0.18 | (-0.30, +0.67) | 0.887 | 1.000 | ns |
| SEMA3B | Forebrain | Late adult | Senior | +0.41 | (-0.12, +0.93) | 0.232 | 0.496 | ns |
| SEMA3B | Hindbrain | Late embryonic | Neonate | +0.19 | (-0.83, +1.21) | 0.993 | 1.000 | ns |
| SEMA3B | Hindbrain | Late embryonic | Juvenile | +0.84 | (+0.01, +1.66) | 0.045 | 0.119 | ns |
| SEMA3B | Hindbrain | Late embryonic | Prime adult | +1.38 | (+0.45, +2.32) | 0.001 | 0.005 | ****** |
| SEMA3B | Hindbrain | Late embryonic | Late adult | +0.98 | (+0.16, +1.80) | 0.011 | 0.041 | ***** |
| SEMA3B | Hindbrain | Late embryonic | Senior | +1.50 | (+0.49, +2.52) | 0.001 | 0.005 | ****** |
| SEMA3B | Hindbrain | Neonate | Juvenile | +0.65 | (-0.50, +1.80) | 0.546 | 1.000 | ns |
| SEMA3B | Hindbrain | Neonate | Prime adult | +1.20 | (-0.03, +2.42) | 0.061 | 0.152 | ns |
| SEMA3B | Hindbrain | Neonate | Late adult | +0.79 | (-0.36, +1.94) | 0.323 | 0.657 | ns |
| SEMA3B | Hindbrain | Neonate | Senior | +1.32 | (+0.02, +2.61) | 0.045 | 0.119 | ns |
| SEMA3B | Hindbrain | Juvenile | Prime adult | +0.55 | (-0.53, +1.62) | 0.646 | 1.000 | ns |
| SEMA3B | Hindbrain | Juvenile | Late adult | +0.15 | (-0.83, +1.13) | 0.998 | 1.000 | ns |
| SEMA3B | Hindbrain | Juvenile | Senior | +0.67 | (-0.48, +1.82) | 0.510 | 0.987 | ns |
| SEMA3B | Hindbrain | Prime adult | Late adult | -0.40 | (-1.47, +0.67) | 0.869 | 1.000 | ns |
| SEMA3B | Hindbrain | Prime adult | Senior | +0.12 | (-1.11, +1.35) | 1.000 | 1.000 | ns |
| SEMA3B | Hindbrain | Late adult | Senior | +0.52 | (-0.63, +1.67) | 0.747 | 1.000 | ns |

## Supplementary Table 4. Source Studies (Accessions) by Brain Region and Developmental Stage

All analyzed libraries are RNA-seq. The table below lists each contributing Bgee experiment/accession with the brain regions and developmental stages it contributes, to document study-of-origin composition and any confounding between stage and source study.

| **Accession** | **Brain region(s)** | **Developmental stage(s) contributed** |
| --- | --- | --- |
| ERP109002 | Forebrain, Hindbrain | Late embryonic, Neonate, Juvenile, Prime adult, Late adult |
| GSE30352 | Forebrain, Hindbrain | Prime adult, Late adult, Senior |
| SRP065273 | Forebrain | Juvenile, Prime adult |
| GSE62098 | Forebrain | Juvenile |
| SRP051844 | Forebrain | Prime adult, Senior |
| SRP034929 | Forebrain | Juvenile, Prime adult |
| GSE43520 | Forebrain | Late adult |
| ERP111066 | Forebrain | Prime adult, Late adult |
| ERP116771 | Forebrain | Prime adult, Late adult |
| SRP015370 | Forebrain | Late adult |
| SRP043364 | Forebrain | Prime adult, Late adult |
| SRP058181 | Forebrain | Late adult, Senior |
| SRP100948 | Forebrain | Senior |
| SRP111096 | Forebrain, Hindbrain | Prime adult, Late adult, Senior |
| SRP216705 | Forebrain | Senior |
| SRP287843 | Forebrain | Prime adult, Late adult, Senior |
| SRP314149 | Forebrain | Late adult, Senior |

*Note: All late embryonic and neonatal samples derive from a single study (ERP109002), which is also the only source contributing paired forebrain and hindbrain tissue across early stages. Because the earliest developmental stages are not distributed across multiple independent studies, developmental stage and study of origin are partially confounded at these stages; formal cross-study batch correction was therefore not applied, and early-stage and hindbrain comparisons are interpreted as exploratory.*
